# Field Testing of Different Chemical Combinations as Odour Baits for Trapping Wild Mosquitoes in The Gambia

**DOI:** 10.1371/journal.pone.0019676

**Published:** 2011-05-26

**Authors:** Musa Jawara, Taiwo S. Awolola, Margaret Pinder, David Jeffries, Renate C. Smallegange, Willem Takken, David J. Conway

**Affiliations:** 1 Medical Research Council Laboratories, Fajara, Banjul, The Gambia; 2 Nigerian Institute of Medical Research, Lagos, Nigeria; 3 Laboratory of Entomology, Wageningen University, Wageningen, The Netherlands; 4 Department of Infectious and Tropical Diseases, London School of Hygiene and Tropical Medicine, London, United Kingdom; Tulane University, United States of America

## Abstract

Odour baited traps have potential use in population surveillance of insect vectors of disease, and in some cases for vector population reduction. Established attractants for human host-seeking mosquitoes include a combination of CO_2_ with L-lactic acid and ammonia, on top of which additional candidate compounds are being tested. In this field study in rural Gambia, using Latin square experiments with thorough randomization and replication, we tested nine different leading candidate combinations of chemical odorants for attractiveness to wild mosquitoes including anthropophilic malaria vectors, using modified Mosquito Magnet-X (MM-X) counterflow traps outside experimental huts containing male human sleepers. Highest catches of female mosquitoes, particularly of *An. gambiae s.l.* and *Mansonia* species, were obtained by incorporation of tetradecanoic acid. As additional carboxylic acids did not increase the trap catches further, this ‘reference blend’ (tetradecanoic acid with L-lactic acid, ammonia and CO_2_) was used in subsequent experiments. MM-X traps with this blend caught similar numbers of *An. gambiae s.l.* and slightly more *Mansonia* and *Culex* mosquitoes than a standard CDC light trap, and these numbers were not significantly affected by the presence or absence of human sleepers in the huts. Experiments with CO_2_ produced from overnight yeast cultures showed that this organic source was effective in enabling trap attractiveness for all mosquito species, although at a slightly lower efficiency than obtained with use of CO_2_ gas cylinders. Although further studies are needed to discover additional chemicals that increase attractiveness, as well as to optimise trap design and CO_2_ source for broader practical use, the odour-baited traps described here are safe and effective for sampling host-seeking mosquitoes outdoors and can be incorporated into studies of malaria vector ecology.

## Introduction

Surveillance of malaria vector mosquitoes is vital for understanding transmission ecology and monitoring changes that occur in response to interventions. Several methods that can be used to sample mosquitoes inside houses are less efficient for outdoor sampling. Human-landing catches have been used for indoor and outdoor sampling of biting populations [1], but there are challenges in standardisation as individual subjects vary in attractiveness to mosquitoes [2], [3] and the process is labour intensive and not widely approved due to safety concerns. Host-seeking female mosquitoes are attracted by carbon dioxide (CO_2_) [4], [5] and odours emanating from sweat and skin [6], [7]. Identification of some of the chemical compounds responsible suggests the possibility of developing safe and standardised odour-baited traps [8].

Of the abundant compounds in human sweat, ammonia and L-lactic acid have been shown to be attractive to female mosquitoes of several species including the principal African malaria vector *Anopheles gambiae sensu stricto* , and other chemicals including carboxylic acids have been tested for effects on top of these basic compounds combined with CO_2_ in an attempt to attract this anthropophilic species [9]. In a recent study to screen 15 different carboxylic acids for attractiveness to colony-bred female *An. gambiae s.s.*, tetradecanoic acid was the only compound to elicit enhanced attraction at all air flow-rates tested in dual-port olfactometer assays [10]. More complex blends derived by using combinations of carboxylic acids did not significantly increase the attractiveness. Importantly, tetradecanoic acid with ammonia and L-lactic acid was also shown to be robustly attractive when delivered within Mosquito Magnet-X (MM-X) counterflow traps in laboratory experiments, as such traps are physically designed for outdoor field use and enable discrimination between the attractiveness of different odour baits [11].

We previously established methods suitable for effective use of MM-X traps in capturing wild mosquitoes within or outside experimental huts in a field site in The Gambia. A series of experiments over a single annual malaria transmission season showed that nylon socks previously worn by volunteer subjects act as reliable positive control attractants in combination with CO_2_, and these enabled us to established optimal placement of the MM-X traps to obtain maximum outdoor mosquito catches [12]. Here, we present assays of different chemical compounds and odour blends for attractiveness in this outdoor trapping system, conducted over the subsequent two annual transmission seasons. Consistent with the laboratory studies, we show that tetradecanoic acid with ammonia and L-lactic acid is the most attractive blend delivered with CO_2_, and none of the additional carboxylic acids tested in more complex blends enhanced the attractiveness. In anticipation of community-based testing of this odour-based trap system we also performed experiments on the use of organic production of CO_2_ by yeast fermentation as an alternative to normal use of an industrial CO_2_ source in cylinders, following initial development of this approach elsewhere [13], [14]. In outdoor trap experiments, we show that the cylinder delivery was more effective, although odour-baited traps with either source of CO_2_ caught equal or larger numbers of mosquitoes than unbaited CDC light traps.

## Methods

### Ethics Statement

This study including the protocols for mosquito trapping and schedule of volunteers sleeping in experimental huts was reviewed and approved by the Gambia Government/MRC Joint Ethics Committee. The illiterate volunteers gave informed consent verbally, as witnessed and documented by the study team. The Ethics Committee approved the consent procedure in this low-risk research project, recognising that the familiarity with and support of the entomology studies at Walikunda by these volunteer men from a neighbouring village is fully testified, and that procedures followed those of earlier studies [12].

### Experimental site

Details of the study area and the experimental huts have been described elsewhere [2], [12]. Briefly, Walikunda is a small fishing village of approximately 50 inhabitants, located in a freshwater region on the south bank of the Gambia River, in the Central River Region of The Gambia. There are six experimental huts that have been purpose-built for studying mosquito behaviour and measuring the efficacy of different interventions. The mean outdoor temperature, relative humidity and rainfall during the period of the experiments were recorded using a weather unit and data logger. A single male adult was employed to sleep in each hut under an intact untreated mosquito net.

### Mosquito Trapping Devices

The nightly trapping experiments started at 21.00 each evening and ended 07.00 the following morning. MM-X counterflow traps manufactured by the former American Biophysics Corporation (ABC, USA) were used, each suspended by a tripod with the trap opening 15 cm above the veranda floor immediately outside experimental huts, a position previously determined to be optimal [12]. These traps were adjusted to hold the chemical blends in sachets as described below, attached to a metal wire within the flow chamber of the trap. CO_2_ was delivered to the trap through silicone tubing attached either to (i) a cylinder containing 100% CO_2_ (prepared by Banjul Oxygen Ltd, Banjul) at a flow rate of 500 ml minute^−1^ controlled through a flow regulator, or (ii) fermentation flasks containing yeast cultures that emitted CO_2_ at an average rate of approximately 200 ml minute^−1^. The cylinder supply of CO_2_ to traps at this experimental site has been described previously [12] and the yeast culture method of CO_2_ production was based on a protocol developed recently [13], [14]. Pictures illustrating MM-X traps in various experimental operations have previously been shown [11], [12], [14]. Our preliminary experiments in the laboratory in The Gambia for local optimisation showed that 2.5 litre reagent bottles containing 200 g sugar, 1 litre tap water and 35 g dried yeast (Pasha, Aruba and Pakmayo brands produced in China were tried) yielded maximal average rates of 50–100 ml CO_2_ minute^−1^ for over 10 hours, consistent with expectations from assays elsewhere [14]. Each of the commercial sources of yeast was effective in the preliminary tests, but the Pakmayo brand was preferred and used in subsequent experiments as it was easily locally available and did not produce excessive froth in the cultures. These conditions were applied outside in the field with the substitution of fresh river water for tap water with similar results, so that employing two bottles in tandem gave rates of CO_2_ flow of 100–250 ml minute^−1^ throughout the 10 hour overnight trapping period. The bottles were positioned within 1 meter of the MMX trap in a large plastic basin to protect from wind or physical accident. For comparison in some experiments, standard CDC miniature light traps (John W. Hock Company, Gainsville, Florida) were also used immediately outside separate huts within a randomised Latin square design.

### Odour blends and delivery

**Figure 1:**
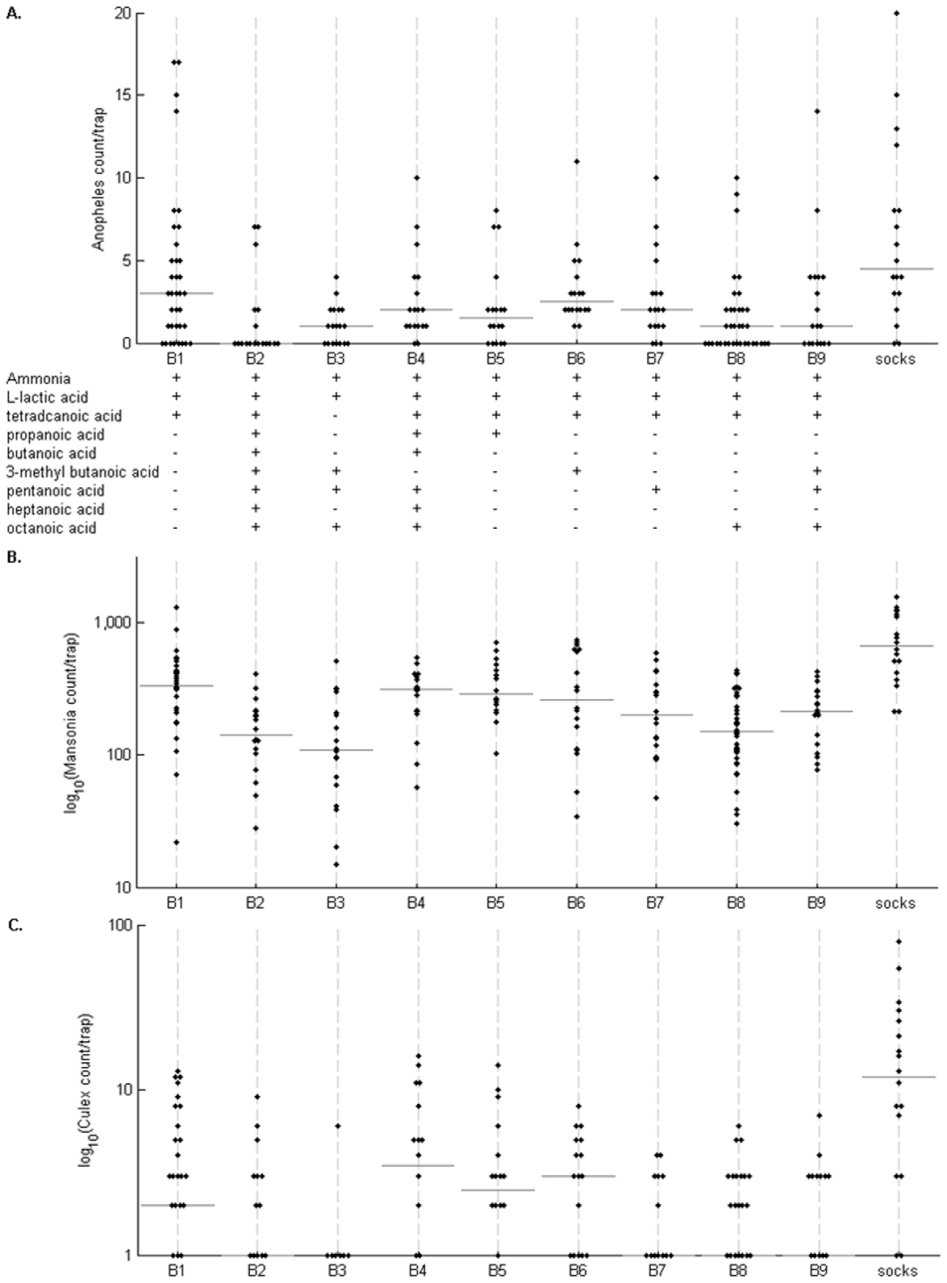
Numbers of mosquitoes captured outside experimental huts in MM-X counter-flow traps. The traps delivered CO_2_ baited with nine different synthetic chemical odour blends (B1 – B9) in comparison with worn socks. (A) Numbers of *An. gambiae s.l.* females per trap-night, with the composition of chemical blends shown underneath, (B) numbers of *Mansonia* sp. mosquitoes per trap-night, (C) numbers of *Culex* sp. mosquitoes per trap-night.

Nine combinations of synthetic odours (designated as blends B1–B9) used in the study were selected from those tested previously in laboratory assays for attractiveness to colony-reared female *Anopheles gambiae s.s* mosquitoes [10]. The primary blend (B1) shown to be robustly attractive in laboratory assays, consisted of ammonia, L-lactic acid, and tetradecanoic acid (the 14-carbon carboxylic acid also known as myristic acid due to its original derivation from nutmeg *Myristica fragrans*), and this combination is considered as the reference blend here. The formulation of the remaining blends tested here involved addition of combinations of 6 different carboxylic acids (propanoic acid, butanoic acid, 3-methyl butanoic acid, pentanoic acid, heptanoic acid, and octanoic acid) to the reference blend, except for blend B3 that did not contain tetradecanoic acid (combination of chemicals forming each blend is shown in Figure 1).

The blends were delivered by slow release of individual volatile chemicals through low density polyethylene (LDPE) square sachets (2.5 cm×2.5 cm; Audion Elektro, The Netherlands), as used in laboratory studies previously [10]. Each chemical was placed in an individual sachet as a liquid of 100 µl volume, or 50 mg crystalline powder in the case of tetradecanoic acid. Ammonia was released using an LDPE sachet of 0.03 mm thickness, and L-lactic acid with an LDPE sachet of 0.05 mm thickness. Each carboxylic acid was released from sachets of 0.1 mm thickness (except for tetradecanoic acid that was released from a sachet of 0.03 mm thickness) in most experiments, after an initial testing of 5 different LDPE thicknesses (0.03, 0.05, 0.1, 0.15 and 0.2 mm) was performed. Each trap contained a blend of chemicals as a combination of different sachets each containing a single chemical, clipped onto a wire carrier in the central trap compartment. A nylon sock worn by a human volunteer (known to be attractive in a previous study that included a panel of local volunteers [12]) was used as a positive control attractant in experiments 1 and 2, following the method previously described [12]. CO_2_ was supplied to each trap from gas cylinders or from yeast cultures through silicone tubing as described above.

### Mosquito Collection and Identification

Each morning, the MM-X traps were stopped by inserting a plug to prevent mosquitoes from escaping, and disconnecting the batteries and switching off the gas regulators at precisely 7.00 am. The windows of the experimental huts were first closed at 6.30 am and the exit traps blocked with a piece of clothing material to prevent mosquitoes that entered from exiting. The MM-X traps were then put in a freezer at −20°C to kill all the mosquitoes, before being emptied for identification. The verandas and rooms were visually searched for live mosquitoes and these were collected by means of a sucking tube / aspirator and placed into labelled cups. A 10 minute search was conducted for each hut (room and veranda). The exit traps were also emptied into a labelled cup and all the mosquitoes were placed in the freezer at −20°C for ∼2 hours before they were subjected to morphological examination and identification. In this area, malaria is transmitted by the *An. gambiae s.l.* complex, and morphological identification was used for principal analysis, as the only two molecular species of the complex occurring at the site are *An. arabiensis* and *An. gambiae sensu stricto* form M [12], [15], and it was necessary to perform immediate analysis of trap data to enable informed design of the series of experiments to fit within short transmission periods (July-September 2008 and 2009). Nuisance biting non-malaria vector species of *Anopheles*, *Culex*, *Aedes*, and *Mansonia* are also common, with mosquitoes of the latter genus being most abundant at the study site. Due to the very large numbers of non-anopheline mosquitoes that are not malaria vectors (sometimes exceeding 1000 per individual trap), these were not identified to species but were identified and analysed by genus.

### Experimental Design and Data Analysis

The experiments were designed using Latin square designs, with randomisation based on a 6 hut x 6 night array that was balanced to control for any carry-over effects of treatments. In some of the experiments Latin squares were combined into a Latin rectangle of 6 huts x 12 nights or larger replicate extensions. Appropriately transformed trap counts were analysed using analysis of variance. Omnibus tests of treatment effect were performed using F-tests and treatment subsets were compared using contrasts. The resulting P values were not adjusted for multiplicity of tests.

## Results

### Comparison of nine synthetic chemical odour blends for attractiveness to wild mosquitoes outdoors

Nine different chemical odour blends were tested outside experimental huts in a large extended Latin square experiment. The blends, B1 – B9 (compositions shown in Figure 1) were tested in comparison with worn socks that had previously been shown to be attractive to mosquitoes under these conditions. Each blend type was randomly allocated outside each hut for 3 nights, giving 18 trap-nights for each blend (and for the worn socks), except for the reference blend B1 that was duplicated in the array and thus tested for 36 trap-nights. L-lactic acid and ammonia were always delivered in sachets of 0.05 mm thickness, while other components were tested in sachets of thicknesses varying from 0.03 to 0.2 mm. A nested analysis was conducted to explore possible effects of varying thickness of LDPE sachets for components of five of the odour blends: (i) B2, (ii) B3, (iii) B5, (iv) B6, and (v) B7. There were no significant differences in numbers of mosquitoes trapped for different LDPE sachet thicknesses for any components of blends B2, B3, B5, and B6, but for pentanoic acid in blend B7 the median LDPE sachet thickness of 0.1 mm gave highest trap numbers of *An. gambiae s.l.* females. The crystalline tetradecanoic acid was subsequently tested in sachets of 0.03 mm thickness, and liquid carboxylic acids including pentanoic acid were subsequently tested in sachets of 0.1 mm thickness.

Comparing all of the nine different chemical blends, there were highly significant differences (P<0.0001). Highest catches of *An. gambiae s.l.* females were obtained from the reference blend B1 (ammonia with L-lactic and tetradecanoic acid), although worn socks gave significantly higher numbers (P = 0.019) (Figure 1A). The reference blend B1 also gave highest catches of *Mansonia* mosquitoes, almost approaching numbers attracted by the worn socks (Figure 1B), whereas *Culex* mosquitoes were not so well attracted by any of the blends in comparison with the worn socks (Figure 1C). Subsequent experiments were conducted on the efficacy of trapping with the reference blend B1.

### Comparison of odour-baited MM-X traps with CDC light traps for outdoor collection

**Figure 2:**
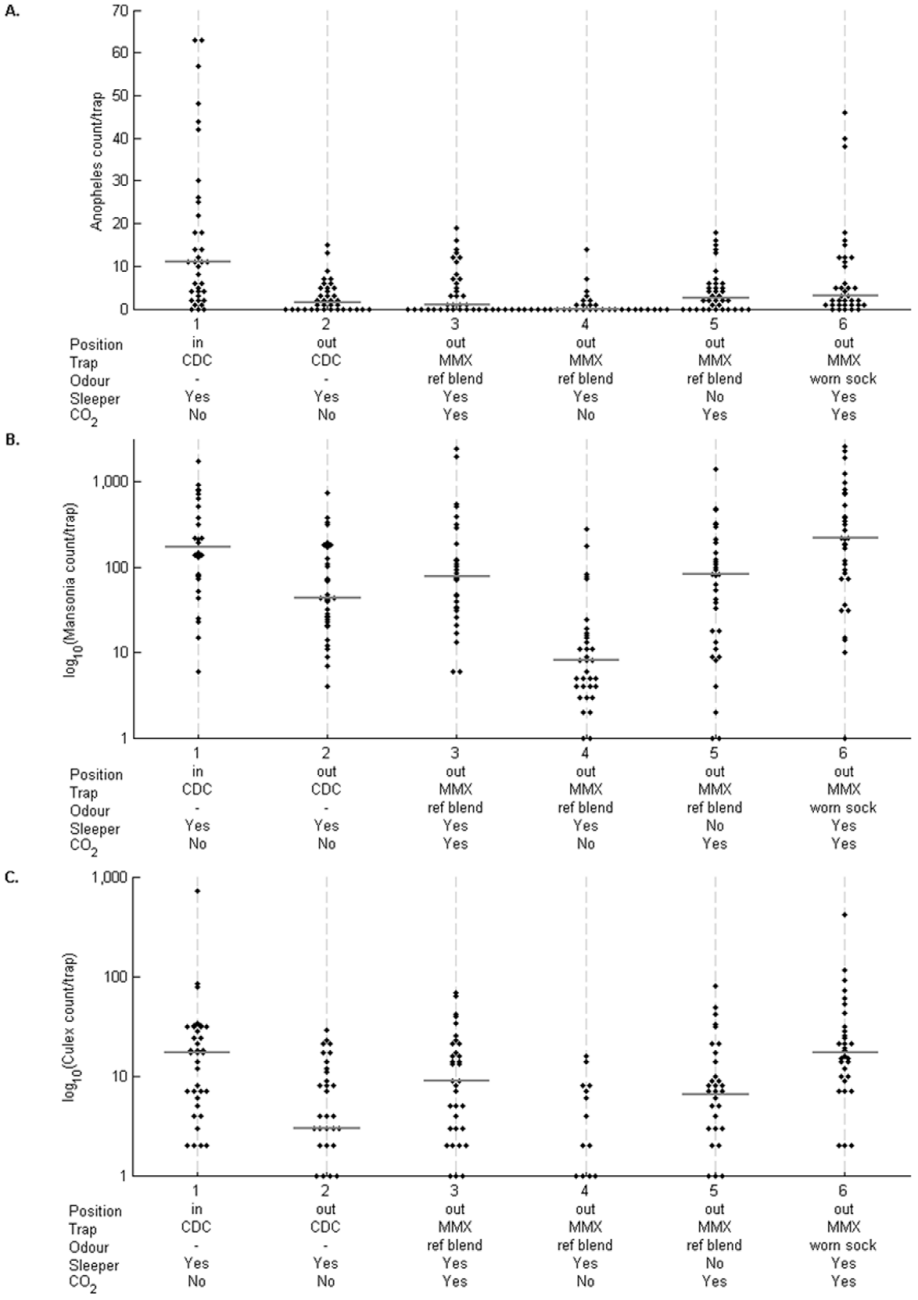
Numbers of mosquitoes captured outside experimental huts in MM-X traps with reference chemical blend B1 or worn socks in comparison with CDC light trap outside or inside. MM-X traps with the reference chemical blend were tested in the presence and absence of CO_2_ and a sleeper in each hut. (A) Numbers of *An. gambiae s.l.* females per trap-night, (B) numbers of *Mansonia* sp. mosquitoes per trap-night, (C) numbers of *Culex* sp. mosquitoes per trap-night.

The next experiment compared the numbers of mosquitoes collected in MM-X traps baited with blend B1 with the numbers collected in standard CDC light traps. The CDC light traps positioned indoors at the foot of the bed with a sleeper captured significantly higher numbers of *An. gambiae s.l.* females than any of the traps positioned outside (Figure 2A). Among the traps placed immediately outdoors on the hut veranda, MM-X traps baited with a worn sock trapped highest numbers as expected, whereas MM-X traps baited with the reference blend B1 caught similar numbers to a CDC light trap (Figure 2A). The numbers caught by the MM-X trap with blend B1 were not significantly affected by the presence of a sleeper in the hut, but were dependent on the use of CO_2_ in the trap. Outdoor catches of *Mansonia* and *Culex* mosquitoes by MM-X traps baited with blend B1 and CO_2_ were slightly higher than catches by CDC light traps (Figure 2B and 2C).

### Use of an alternative source of CO_2_

Experiments were next conducted on the use of CO_2_ produced organically from overnight yeast cultures, as a potential alternative to using CO_2_ from gas cylinders for the MM-X traps. Initial experiments in the laboratory at Fajara in The Gambia over a two week period prior to field testing indicated that dual flasks usually led to an average production of ∼200 ml min^−1^ over a 10 hour period overnight at ambient temperatures, while use of a single flask culture generally delivered approximately half this amount as expected. Use of larger cultures was not logistically convenient, so this dual flask system was compared directly to the use of gas cylinder CO_2_ using MMX-traps baited with blend B1 outside experimental huts, using a Latin square design for 6 huts over 6 nights. The mean CO_2_ production from the outdoor yeast cultures measured at 6 sampling times over the 10 hour collection period over each of the first 2 nights was 180 ml min^−1^, a value similar to that measured in the laboratory.

**Figure 3:**
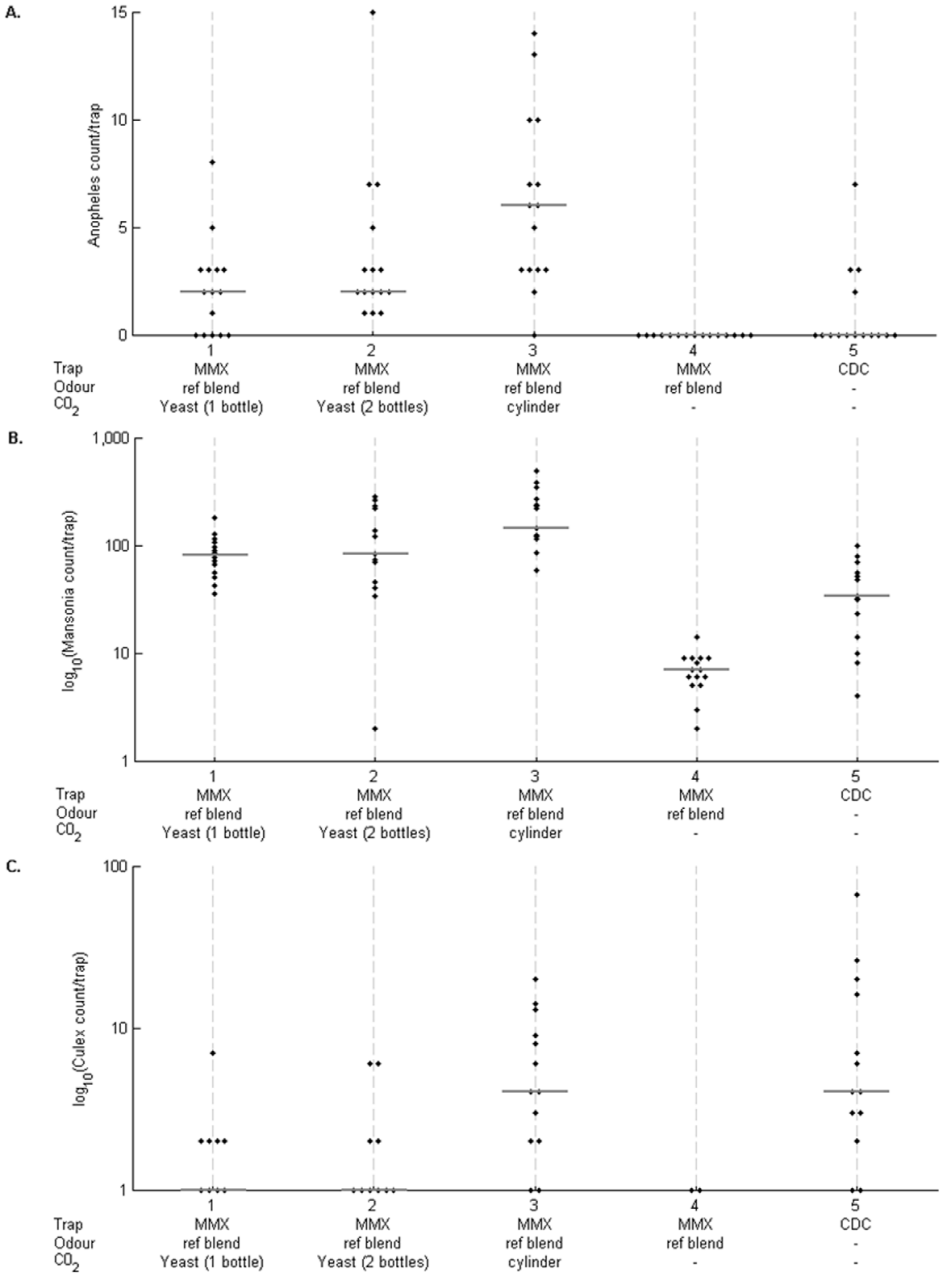
Numbers of mosquitoes captured outside experimental huts in MM-X traps with reference chemical blend B1 and CO_2_ from cylinders or yeast cultures or no CO_2_ in comparison with CDC light traps. (A) Numbers of *An. gambiae s.l.* females per trap-night, (B) numbers of *Mansonia* sp. mosquitoes per trap-night, (C) numbers of *Culex* sp. mosquitoes per trap-night.

As expected, the MM-X trap without CO_2_ yielded virtually zero catches except for *Mansonia* mosquitoes, so this was dropped from the analysis of variance to enable more power for the comparisons of the other traps. The catch numbers of *An. gambiae s.l.* in the traps using yeast-derived CO_2_ were slightly higher when two flasks were used rather than one, but the numbers were less than were obtained with traps using cylinder-derived CO_2_. This difference was approximately 2-fold for *An. gambiae s.l.* females, traps with cylinder supply capturing significantly more than single-flask traps (P = 0.0005) although not statistically significantly more than double-flask traps (P>0.05)(Figure 3A). For the other major mosquito genera, catch numbers were significantly higher in the traps supplied by cylinder compared with both the traps with single- and double-flask yeast CO_2_ (P = 0.0001 and 0.0043 respectively for *Mansonia*, P = 0.014 and 0.023 respectively for *Culex*; Figure 3B and 3C).

## Discussion

The present study shows that a simple blend of only 3 chemicals (L-lactic acid, ammonia, and tetradecanoic acid) delivered together with CO_2_ in a counterflow trap, is equally or more attractive to wild mosquitoes including *An. gambiae s.l.* than more complex blends with additional components. This attractiveness was robust and reproducible, approaching but not quite matching that obtained by using worn socks as human odour bait. This is in agreement with the relative attractiveness shown in olfactometer experiments previously conducted under laboratory conditions with colony-reared mosquitoes [10]. A recent claim that one of the more complex chemical blends may be more attractive than human odour [16] is not supported here, nor by other experiments that incorporate a high level of control and replication [17]. Further research to discover chemical odours that mosquitoes detect in host-seeking, including receptor characterisation [18] with a rigorous pipeline of validated assays based on electrophysiological [19] and behavioural experiments [10], [20], is needed to potentially add to the attractiveness of the existing reference blend.

Although the optimal reference chemical blend does not achieve trap catches as high as those elicited by human odour, it has the distinct advantage of standardisation for use in traps for ecological sampling over space and time. With the scale up of indoor interventions against mosquito vectors in Africa, particularly the use of ITNs and IRS, and decreasing transmission of malaria in some countries including The Gambia [21], outdoor biting mosquitoes are likely to become more important in maintaining residual transmission of malaria. In comparison with the standard CDC light trap, the odour-baited trap performed favourably for outdoor trapping here, and it will be important to evaluate its potential as a standardised trap for sampling mosquitoes outside houses.

Although the yeast-derived CO_2_ source was effective in making the traps attractive, it was not as powerful as the CO_2_ cylinders that could routinely deliver a higher flow rate. Increasing the culture bottle size and the amount of yeast or sugar could increase the CO_2_ production [14], but the larger and more cumbersome operation would be less appealing for field use. Moreover, the cylinder CO_2_ delivery was more standardised, and took less time to set up for each trap-night. Therefore, where cylinders can be safely transported and left in a location for repeated sampling, we consider that their use is generally preferable to the yeast culture method, and could be applied to mosquito sampling in some rural villages in The Gambia. However, where procurement and safe transport of gas cylinders is very difficult, and other methods of CO_2_ generation including use of dry ice are also impracticable [22], the use of yeast-derived CO_2_ provides a potential alternative. Utilising either source of CO_2_, it would be worth piloting these chemical odour-baited traps for surveillance of mosquitoes outside houses, recognising that a method of choice depends on local logistics and costs as well as on absolute levels of attractiveness to mosquitoes.
